# High Levels of Prebiotic Resistant Starch in Diet Modulate a Specific Pattern of miRNAs Expression Profile Associated to a Better Overall Survival in Pancreatic Cancer

**DOI:** 10.3390/biom11010026

**Published:** 2020-12-29

**Authors:** Nadia Trivieri, Concetta Panebianco, Annacandida Villani, Riccardo Pracella, Tiziana Pia Latiano, Francesco Perri, Elena Binda, Valerio Pazienza

**Affiliations:** 1 Cancer Stem Cells Unit, ISBReMIT, Fondazione IRCCS “Casa Sollievo della Sofferenza”, viale Padre Pio, 7-71013 San Giovanni Rotondo, 71100 Foggia, Italy; n.trivieri@css-mendel.it (N.T.); riccardopracella@gmail.com (R.P.); 2 Gastroenterology Unit, Fondazione IRCCS “Casa Sollievo della Sofferenza” Hospital, viale dei Cappuccini, 1-71013 San Giovanni Rotondo, 71100 Foggia, Italy; panebianco.c@gmail.com (C.P.); a.villani@operapadrepio.it (A.V.); f.perri@operapadrepio.it (F.P.); 3 Oncology Unit, Fondazione IRCCS “Casa Sollievo della Sofferenza” Hospital, viale dei Cappuccini, 1-71013 San Giovanni Rotondo, 71100 Foggia, Italy; latianotiziana@gmail.com

**Keywords:** prebiotics, resistant starch, miRNAs, pancreatic cancer

## Abstract

Dietary patterns are well known risk factors involved in cancer initiation, progression, and in cancer protection. Previous in vitro and in vivo studies underline the link between a diet rich in resistant starch (RS) and slowing of tumor growth and gene expression in pancreatic cancer xenograft mice. The aim of this study was to investigate the impact of a diet rich in resistant starch on miRNAs and miRNAs-target genes expression profile and on biological processes and pathways, that play a critical role in pancreatic tumors of xenografted mice. miRNA expression profiles on tumor tissues displayed 19 miRNAs as dysregulated in mice fed with RS diet as compared to those fed with control diet and differentially expressed miRNA-target genes were predicted by integrating (our data) with a public human pancreatic cancer gene expression dataset (GSE16515). Functional and pathway enrichment analyses unveiled that miRNAs involved in RS diet are critical regulators of genes that control tumor growth and cell migration and metastasis, inflammatory response, and, as expected, synthesis of carbohydrate and glucose metabolism disorder. Mostly, overall survival analysis with clinical data from TCGA (*n* = 175) displayed that almost four miRNAs (miRNA-375, miRNA-148a-3p, miRNA-125a-5p, and miRNA-200a-3p) upregulated in tumors from mice fed with RS were a predictor of good prognosis for pancreatic cancer patients. These findings contribute to the understanding of the potential mechanisms through which resistant starch may affect cancer progression, suggesting also a possible integrative approach for enhancing the efficacy of existing cancer treatments.

## 1. Introduction

Pancreatic cancer (PC) is recognized to be one of the most aggressive and lethal malignancies with a dismal prognosis considering that less than 5% for patients with a surgically resectable disease will survive up to 5 years [1] while for patients with advanced metastasized disease an average survival time of 6 months is expected [2]. Unhealthy diet, high body mass index, tobacco use, alcohol abuse, and physical inactivity are the major cancer risk factors according to WHO data [3]. Nowadays, many reports have paid particular attention to the topic of nutritional support for pancreatic cancer patients in order to ameliorate the clinical outcome [4] as nutritional elements can powerfully modulate cells and cancer behavior through the regulation of cancer promoting/preventing pathways [5]. Moreover, in order to reduce side effects of the tumor and/or therapeutic treatments, (i.e., malnutrition and metabolic derangements) cancer patients are often encouraged to adopt high protein diet, maintaining caloric intake [6]. Yet, these guidelines do not provide a tangible therapeutic benefit.

Although fasting, fasting-mimicking diet (FMD), calories restriction, and ketogenic diets are dietary interventions already deemed in clinical trials for cancer therapy [7], they not only cause body weight loss but they also require a strong mental discipline [8]. In order to improve the adherence to these “low-calories” regimens, several dietary approaches for cancer treatment, in which well-defined nutrients are removed according to the inherent metabolic requirements of the tumor, are under development. To date, the latter strategies are deemed to be more targeted approaches based also on the tumor’s type. To a greater extent, owing to the increased understanding on cancer metabolism, several types of cancer cells have been shown to display altered glucose metabolism, thus leading to a high uptake of glucose as compared to adjacent normal tissues. More thoroughly, glucose is a well-known major source of energy for most cells and glycolysis supports the rapid proliferation and redox defense in cancer cells [9]. Ordinary mice diets contain corn starch (CS) as carbohydrates substrate which are metabolized in the small intestine to release glucose. Yet, when CS is replaced by resistant starch (RS), which instead gets to the large bowel undigested, glucose is no longer released nor absorbed into the blood. There exist different types of RS, of which some are naturally occurring in foods such as whole grains and seeds, legumes, row potatoes, green bananas, other produced after cooking, as cooked and cooled potatoes, cornflakes, high-amylose maize starch, and finally others obtained by chemical modification [10,11]. RS consumption is increasingly deemed to confer health benefits, due to several mechanisms including low glycemic index, improved blood lipid profile, prebiotic function and shaping of gut microbiota, with consequent production of fermentation products, above all short chain fatty acids (SCFAs) [11]. By virtue of these effects of RS on gut milieu, most preclinical studies have focused on colorectal cancer (CRC), demonstrating RS ability to increase SCFAs and especially butyrate levels, reduce inflammation and intestinal epithelial cells proliferation [12,13], induce apoptosis of damaged colonic epithelium [14], modulate the cancer-related WNT pathway [15]. The beneficial role of RS in clinical studies on CRC patients, however, remain controversial. RS supplementation to diet in healthy volunteers was shown to significantly decrease colonic mucosal proliferation [16] and to protect from red meat-induced DNA adducts in human rectal tissues [17]. On the other hand, two larger CAPP (Concerted Action Polyp Prevention) studies were carried out, aimed to assess the clinical efficacy of RS on CRC prevention. The CAPP1 study conducted on patients with Familial Adenomatous Polyposis failed to demonstrate any detectable effect of RS on number of polyps [18] and the CAPP2 trial on patients with Lynch syndrome did not succeed to observe any preventive effect on the incidence of colorectal adenoma or carcinoma [19]. 

It has been described that nutrients and food compounds are capable of modulating gene expression, also as a result of epigenetic modifications including miRNA regulation [20], and previous reports clearly showed the protective effect of RS-induced modulation of miRNA profile against cancer [21,22].

In our previously published studies, we already demonstrated that diet with high content of RS affects the growth of pancreatic cancer and gut microbiota composition in xenografted mice [23]. 

In more detail, at the end of the treatment, mice fed with engineered resistant starch diet (ERSD) showed statistically significant reduction of tumor volume as compared to control mice, indicating tumor growth retardation. This was accompanied by a significantly decreased expression of Ki67 at the mRNA and protein level in the tumor tissue of ERSD-fed mice with respect to control group [23].

Moreover we demonstrated that RS, like other nutrients, modulate gene expression and metabolism, potentially impacting the progression of cancer disease [24]. In this study, we explore in pancreatic cancer xenograft mice the effect of an engineered resistant starch diet (ERSD) with a high content of RS, as compared to normal diet, on miRNA expression. miRNAs profile was integrated with data from a public mRNA microarray dataset and functional, pathways, and overall survival analyses were conducted on miRNAs and miRNAs target gene pairs to pinpoint the putative impact of ERSD in pancreatic cancer signaling and clinical outcome.

## 2. Materials and Methods

### 2.1. Animal Study

In vivo studies were conducted in strict accordance with the Guidelines for the Care and Use of Laboratory Animals and according to protocols approved by the Institutional Animal Care and Use Committee of the GenScript’s (approval number ANM14-002/468862). As reported in our previous study [23], six weeks old female Nu/Nu nude mice housed under pathogen-free conditions were subcutaneously injected with 5 × 10^6^ BxPC-3-luc tumor cells suspended in 0.1 mL of PBS/matrigel mixture (1:1) per mouse. BxPC-3-luc tumor-bearing nude mice were randomly assigned into two groups (*n* = 6 mice/group): group 1 (standard diet) and group 2 (engineered resistant starch diet, ERSD) with free access to water. Details on ERSD are reported in [23].

### 2.2. RNA Extraction

Total RNA (including miRNA) was extracted from frozen cancer tissues by using the miRNeasy mini kit (Qiagen, Hilden, Germany, Cat # 217004) following the manufacturer’s instructions. RNA concentration was assessed using the NanoDrop 1000 Spectrophotometer (NanoDrop Technologies, Berlin, Germany). Quality control analysis of the isolated samples was evaluated by the Agilent 2100 Bioanalyzer (Agilent Technologies, Waldbronn, Germany).

### 2.3. MicroRNAs and mRNA Analysis 

MicroRNAs expression profiling was performed using the Affymetrix GeneChip miRNA Array 4.0 (Affymetrix Inc., Santa Clara, CA, USA, Cat # 902446) according to the manufacturer’s instructions. For each sample, 150 ng of total RNA was labeled using Flag Tag Biotin HSR labeling. The process began with a brief tailing reaction followed by ligation of the biotinylated molecule to target RNA sample. The Biotin-labeled RNA samples were then hybridized on Affymetrix^®^ 450 Fluidics Station with continuous agitation at 60 rpm for 16 h at 48 °C. The miRNA microarray chips were washed and stained using the Fluidics Station 450 (Affymetrix Inc., Santa Clara, CA, USA) and finally scanned by GeneChip Scanner 3000 7G. In accordance with Affymetrix manuals, raw data (.CEL file) quality control examination was performed by the Expression Console version 1.4.1 (Affymetrix Inc., Santa Clara, CA, USA).

MicroRNA expression profile was then analyzed by the Partek Genomics Suite package ver. 6.6 (Partek, Chesterfield, MO, USA). Expression values were extracted and normalized from raw data (.CEL file) using the *GCRMA* of Partek. Batch effects were removed by the *Partek’s batch effect removal* algorithm. Comparative analysis was conducted between the test sample and the normal control and the differentially expressed miRNAs were called on a minimum log_2_ fold change of 1.3. Principal component analysis (PCA) and hierarchical cluster represented miRNAs showing the highest variance in expression level between the test and normal samples. MicroRNAs data were integrated with mRNA expression raw data (CEL file) downloaded from the public human pancreatic cancer gene expression GSE16515 dataset [25] and differential gene expression analysis between normal and pancreatic cancer tissue were performed by Partek Genomics Suite package ver. 7.0 (Partek, Chesterfield, MO, USA). Normalization was done using the *GCRMA* of Partek. 

### 2.4. Prediction of miRNAs Targets and Functional Enrichment Analysis

In silico analysis of the biological functions, canonical pathways, and regulatory networks in which differential expression of miRNAs were involved was conducted using the ingenuity pathway analysis (IPA; QIAGEN, http://www.ingenuity.com/) and R software. For miRNAs-targets analysis, we used IPA’s microRNA Target Filter, which includes filtering tools that sort microRNA targets and allow to examine microRNA-mRNA pairing. List of miRNAs and mRNAs differentially expressed were filtered for miRNA targets highly predicted and experimentally observed and then analyzed based on the IPA library of canonical pathways. The significance of the association between each list and canonical pathways was measured by Fisher’s exact list. To identify biological functions, right-tailed Fischer’s exact test was used and a significant *p*-value for each category was calculated. The obtained *p*-value was further adjusted using Benjamini–Hochberg false discovery rate (FDR) correction. Cutoff for significance was set as adjusted *p*-value < 0.05, z-score > 2 (minimum activation threshold), and z-score < −2 (minimum inhibition score). Over- and down-represented functions and pathways were represented as forest plots.

### 2.5. Immunoblotting

Proteins were extracted from snap-frozen pancreatic cancer tissues, separated by SDS-PAGE and blotted onto PVDF membranes as previously described [26]. Membranes were incubated overnight at 4 °C with primary antibodies as follows: anti-phospho-PI3K (Tyr458) (1:1000, Cell Signaling Technology, Cat # 4228), anti-PI3K (1:1000, Cell Signaling Technology, Cat # 4257), anti-AKT (1:1000, Santa Cruz Biotechnology, Cat # sc-5298), anti-phospho-mTOR (Ser2481) (1:1000, Cell Signaling Technology, Cat # 2974), anti-mTOR (1:1000, Cell Signaling Technology, Cat # 2972), anti-phospho-p70S6K (Thr389) (1:1000, Cell Signaling Technology, Cat # 9205), anti-p70S6K (1:1000, Cell Signaling Technology, Cat # 9202). Membranes were then incubated with respective anti-rabbit or anti-mouse horseradish-peroxidase-conjugated secondary antibodies (1:3000, Bio-Rad, Cat # 1706515 and 1706516, respectively) and bands were detected with enhanced chemo-luminescence (Thermo Fisher Scientific, Cat # 32132). 

### 2.6. Survival and Statistical Analyses

MicroRNAs expression data and the corresponding clinical information for Pancreatic Adenocarcinoma (PAAD) dataset were downloaded from The Cancer Genome Atlas (TCGA) data portal. From the PAAD dataset, we selected the 175 patients with overall survival (OS) information and level 3 data. Optimal cutoffs between high and low miRNA expression groups were determined through the R package “survminer”. Mantel–Cox and Gehan–Breslow–Wilcoxon tests for groups overall survival comparison were performed by GraphPad Prism, which was used also to plot the Kaplan–Meier curves. A *p*-value < 0.05 was considered statistically significant.

Differential miRNAs and genes target expression from microarray data and from the public human pancreatic cancer gene expression GSE16515 dataset were assessed by the implementation of the ANOVA test available in Partek Genomic Suite 6.6 and 7.0 software version (Partek Inc., Chesterfield, MI, USA). The Benjamini–Hochberg false discovery rate (FDR) was employed to adjust the *p*-values. Only genes with a *p*-value < 0.05 were considered differentially expressed.

## 3. Results

### 3.1. Differential miRNAs and miRNAs Target Genes’ Expression in PC Xenograft Mice under RS Diet

**Figure 1:**
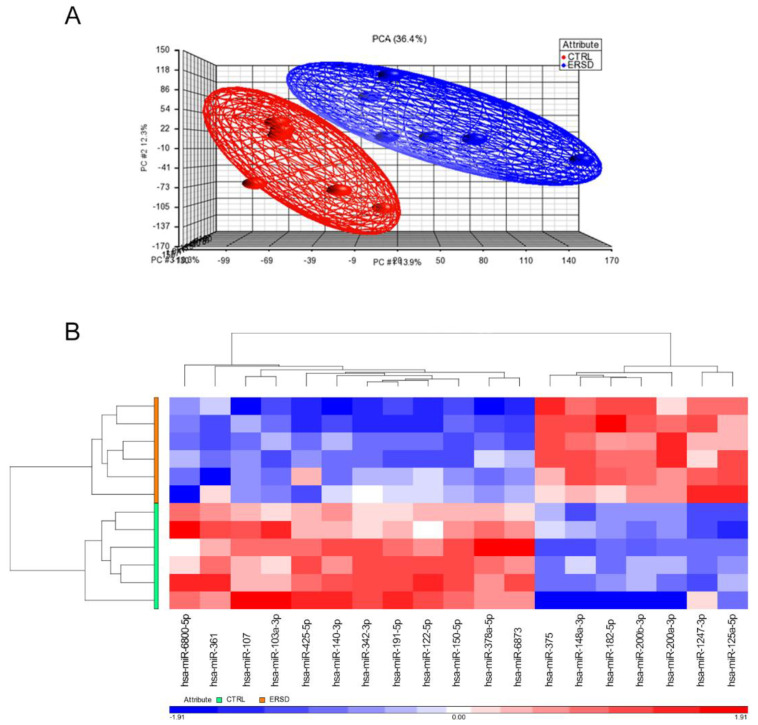
(**A**) Global views of miRNA expression by principal component analysis (PCA) between pancreatic cancer mice fed with the control diet (CTRL, red) and those fed with ERSD (ERSD, blue). Each dot represents a sample and each color the type of the sample. PCA percent values indicate the explained variability on the coordinates. (**B**) Heatmap of hierarchical clustering of 19-identified miRNAs differentially expressed in pancreatic cancer tissue from mice fed with high level of prebiotic resistant starch (ERSD) diet compared to mice fed with normal diet (CTRL). Columns show miRNAs and rows tissue specimens. A dual-color code represents miRNAs up- (red) and downregulated (blue), respectively.

We first analyzed and compared miRNAs expression profiles of pancreatic cancer tissue samples from mice fed with control diet versus mice fed with ERSD by Affymetrix GeneChip miRNA Array v. 4.0. As expected, principal component analysis (PCA) displayed that miRNA profiling clustered samples obtained from control versus ERSD fed mice (Figure 1A). Hierarchical clustering analysis based on the global miRNA expression depicted in Figure 1B clearly distinguished PC mice fed with ERSD to those fed with control nutriment. Among the miRNAs peculiarly enriched in the former and, thus, downregulated in mice fed with ERSD, miRNA-6800-5p, miRNA-361, miRNA-107, miRNA-103a-3p, miRNA-425-5p, miRNA-140-3p, miRNA-342-3p, miRNA-191-5p, miRNA-122-5p, miRNA-150-5p, miRNA-378a-5p, and miRNA-6873 were found. Among these, miR-361-3p, miR-425-5p, miR-191, and miR-122-5p were previously reported to be promoted or to be associated with pancreatic cancer progression, invasion, and metastasis [27,28,29,30]. 

Meanwhile, miRNA-375, miRNA-148a-3p, miRNA-182-5p, miRNA-200b-3p, miRNA-200a-3p, miRNA-1247-3p, and miRNA-125a-5p resulted significantly upregulated in mice fed with ERSD as compared to control mice. These miRNAs, too, have previously been described as involved in protecting from pancreatic cancer development, progression, and/or as being associated to a better prognosis [31,32,33,34,35,36,37,38].

From the observed differential miRNA expression profile between tumor tissues from ERSD and control fed mice, we finally predicted putative targets of differentially expressed miRNAs. We downloaded and integrated our microRNAs expression data with the human pancreatic gene expression dataset GSE16515 [25] from the Expression Ominibus database (GEO) [39]. Two normal tissue samples and 11 pancreatic cancer patients were included and analyzed by Partek Genomic Suite v. 6.6 software to identify differential gene expression between pancreatic cancer and normal tissues. In order to predict miRNA target genes in pancreatic cancer samples [40] we then used ingenuity pathways analysis (IPA) software and obtained 208 miRNA-target gene pairs with an inverse correlation of expression (Table S1). A total of 112 and 86 miRNA-target gene pairs were identified for, respectively, the upregulated and downregulated miRNA in tumor tissues from ERSD and control fed mice (Table S1).

### 3.2. Biological Function and Pathway Enrichment Analysis of miRNA-Target Genes 

**Figure 2:**
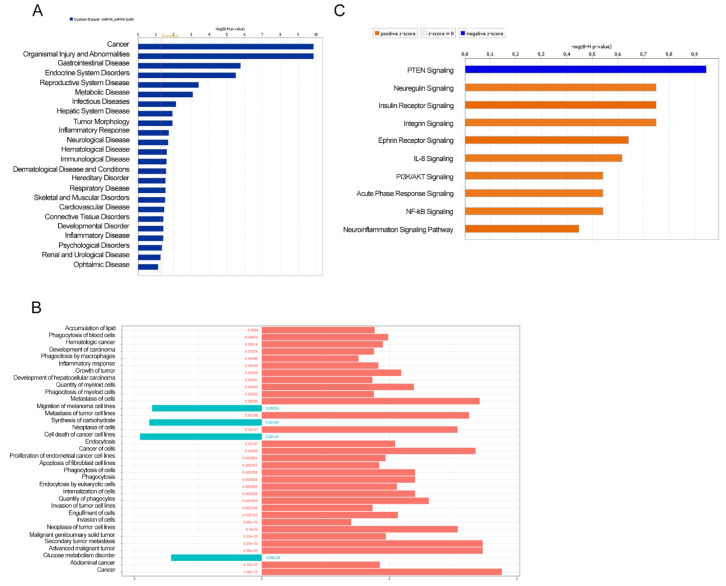
Ingenuity pathways analysis (IPA) illustrating the enriched biological functions in miRNA-target genes of pancreatic cancer tissues of mice fed with control (blue and orange bars in (**A**,**B**), respectively) or ERSD (light blue bars in (**B**)). Significance was calculated by Fisher’s exact test *p*-value. FDR adjusted *p*-values are shown in (**B**). (**C**) The top 10 enriched pathway related to miRNA-target gene pairs in pancreatic cancer tissues of mice fed with ERSD (negative z-score; blue bar) or control diet (positive z-score; orange bar).

To determine the biological function of the differentially enriched miRNA-target genes between mice fed with ERSD as compared to control mice, we next performed predictive analysis by ingenuity pathways analysis (IPA). Gene Ontology (GO) classification of enriched biological functions depicted in Figure 2A,B shows that miRNA-target genes associated with the downregulated miRNAs in tumor tissues from ERSD versus control fed mice were mainly involved in biological processes such as cancer and development of carcinoma, inflammatory response, abdominal cancer, and metabolic disease, growth, invasion, and metastasis of tumor (Figure 2A,B and Table S2). Notably, miRNA-target genes associated to the enhanced miRNA in tumor tissues from ERSD versus control fed mice, were functionally enriched in biological processes including synthesis of carbohydrate, glucose metabolism disorder, and cell death of cancer cell lines (Figure 2B and Table S2).

**Figure 3:**
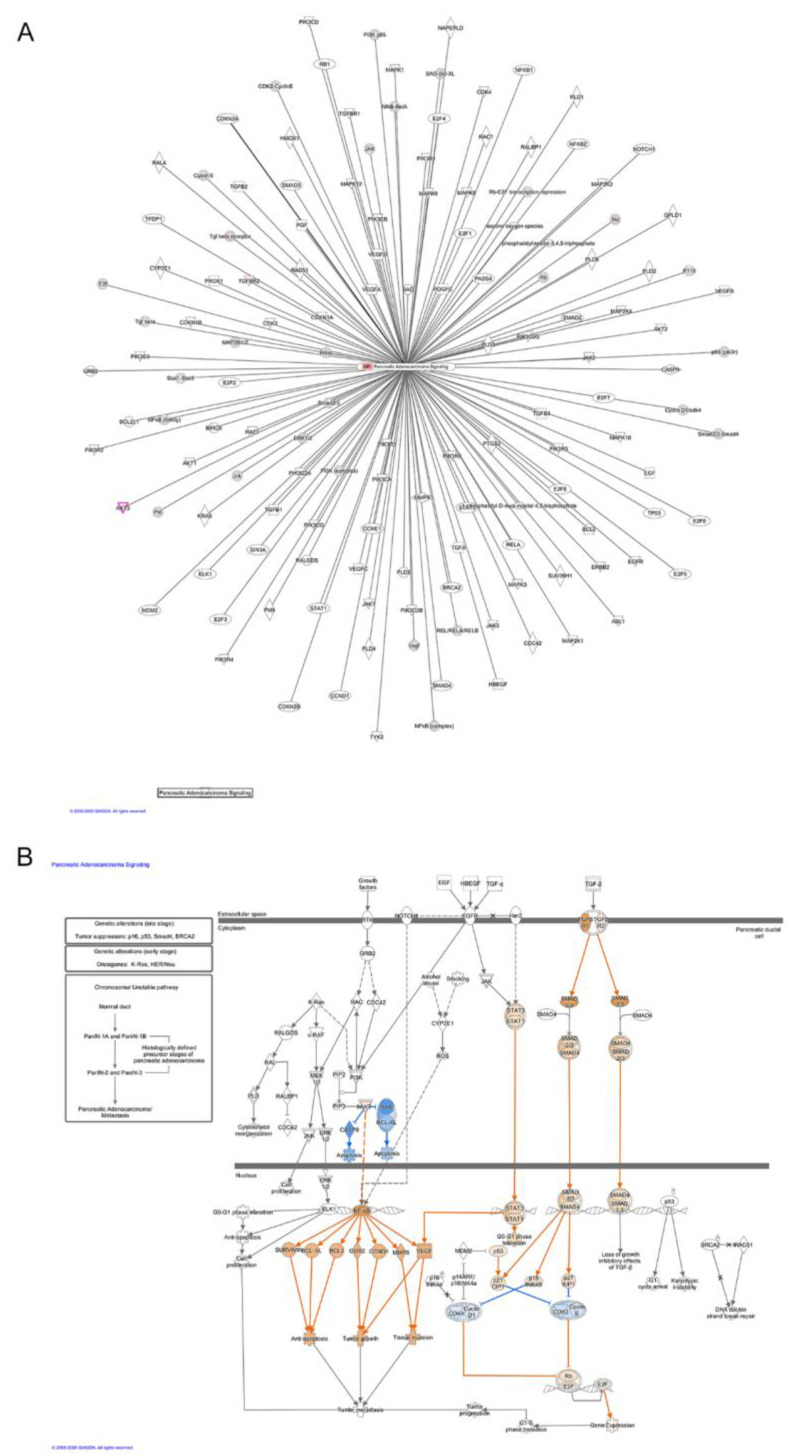
Pathway enrichment analysis. (**A**) Schematic pancreatic adenocarcinoma signaling network generated from IPA analysis based on differentially miRNA-target genes in mice bearing pancreatic tumor and fed with ERSD versus normal nutriment. Upregulated genes, as revealed by fold-change analysis, are red colored. Color intensity indicates the score of upregulation. (**B**) IPA analysis depicting that either NF-kB or TGFβ pathways are activated in pancreatic cancer tissues of mice fed with control diet, leading to the stimulation of tumor growth and invasion, gene expression, and loss of growth inhibitory effects of TGFβ. Genes predicted to be activated (orange-colored) and inhibited (blue-colored) when comparing miRNA-target genes of mice fed with ERSD versus control diet are shown. Color intensity indicates higher absolute z-score.

Moreover, when miRNA-target gene pairs were analyzed for the prediction of canonical pathway enrichment by IPA, only PTEN signaling (Fisher’s exact test, adjusted *p*-value < 0.05) emerged enriched in ERSD miRNA-target genes versus controls fed mice, whereas nine pathways overrepresented among upregulated target genes, included Ephrin Receptor, PIK3/AKT, NF-kB, and IL-8 signaling together with other proinflammatory pathways in pancreatic cancer mice fed with control diet (Figure 2C and Table S3). Specifically, IPA network analysis of pancreatic adenocarcinoma signaling revealed that a total of two miRNA-target genes, being *TGFBR* and *AKT,* were upregulated in control versus ERDS fed mice (Figure 3A). Furthermore, as shown by the IPA pathway network prediction depicted in Figure 3B, NF-kB and TGFBR2 signaling pathways were activated in tumors from control but not in tumors from ERSD fed mice, thus resulting in the stimulation of tumor growth, progression, invasion and metastasis, and gene expression. 

### 3.3. ERSD Influence on PI3K/AKT Signaling

**Figure 4:**
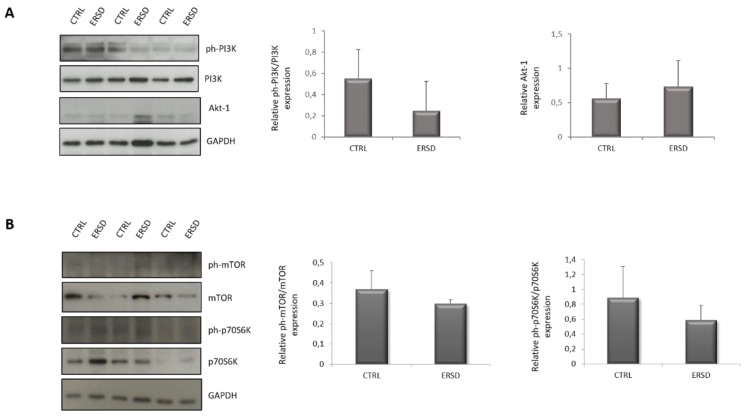
Immunoblot detection and quantification of relative phospho-PI3K normalized to total PI3K protein expression and total AKT protein expression (**A**), relative phospho-mTOR normalized to total mTOR protein expression and relative phospho-p70S6K normalized to total p70S6K protein expression (**B**) in pancreatic cancer tissues of mice fed with control and ERSD. Results are expressed as means ± SD.

To further investigate the influence of ERSD on the PI3K/AKT pathway, which was predicted to be suppressed in comparison to control diet, we assessed the activation status of PI3K, AKT, mTOR, and p70S6K in pancreatic cancer tissues from both experimental groups. As shown in Figure 4, the phosphorylation of PI3K (Figure 4A), mTOR and its downstream target p70S6K (Figure 4B) tended to decrease in ERSD when compared to control diet, though not reaching statistical significance. AKT phosphorylation was not detectable in all samples (not shown), likely due to the low levels of basal AKT activation which are characteristic of BxPC-3 pancreatic cell line [41].

### 3.4. Expression of Four miRNAs in ERSD Predicts Survival of Pancreatic Cancer Patients

**Figure 5:**
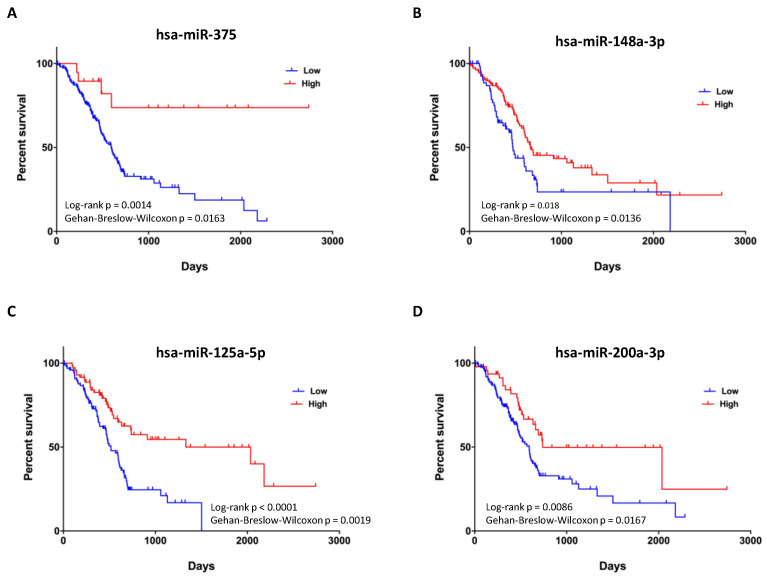
Prognostic value of upregulated miRNAs in pancreatic cancer tissues of mice fed with ERSD versus normal diet. (**A**–**D**) Kaplan–Meier survival curves for 175 pancreatic cancer patients from TCGA dataset revealing that high expression levels of miR-375 (total High, *n* = 19; total Low, *n* = 156), miR-148a-3p (total High, *n* = 110; total Low, *n* = 65), miR-125a-5p (total High, *n* = 75; total Low, *n* = 100), and miR-200a-3p (total High, *n* = 48; total Low, *n* = 127) are associated with a better clinical outcome. *p*-values from log-rank Mantel–Cox and Gehan–Breslow–Wilcoxon tests are indicated.

Finally, to determine whether the 19 miRNAs identified as differentially expressed by our analysis in tumor tissues from ERSD as compared to controls fed mice are associated with pancreatic cancer patients’ prognosis, we evaluated their prognostic performance on a cohort of patients from the TCGA dataset. Analysis from TCGA pancreatic cancer dataset revealed that four miRNAs upregulated in pancreatic cancer mice under ERSD as compared to their normal diet fed mice counterpart, being miRNA-375, miRNA-148a-3p, miRNA-125a-5p, and miRNA-200a-3p, displayed a significant difference in expected clinical outcome of pancreatic cancer patients (Figure 5). Yet, Kaplan–Meier survival analysis for 175 pancreatic cancer patients revealed that subjects characterized by high miR-375 expression were associated with a better outcome than those with low levels (*p* = 0.0014 by log-rank Mantel–Cox test and *p* = 0.0163 by Gehan–Breslow–Wilcoxon test) (Figure 5A) and that a median survival of 666 days versus 473 days pertained cases with miR-148a-3p upregulation as compared to the downregulated counterpart (*p* = 0.018 by log-rank test and *p* = 0.0136 by Gehan–Breslow–Wilcoxon test) (Figure 5B). Furthermore, a median survival of 2036 days versus 517 days distinguished patients with high miR-125a-5p expression versus their low expression counterpart (*p* < 0.0001 by log-rank test and *p* = 0.0019 by Gehan–Breslow–Wilcoxon test) (Figure 5C) and, finally, a better clinical outcome was associated to high levels of miR-200a-3p versus their low counterpart (median survival of 738 and 592 days, respectively; *p* = 0.0086 by log-rank test and *p* = 0.0167 by Gehan–Breslow–Wilcoxon test (Figure 5D).

## 4. Discussion

Public education and information must focus their effort in promoting to adopt a healthy lifestyle in order to prevent or modify the noncommunicable diseases’ outcome including cancer. Since diet is an easily modifiable factor and due to its capacity to modify several features of human biology (including metabolism, immunity, gene expression, and microbiota), nutrients contained within the food we ingest represent one of the key elements influencing our health status [42]. Notably, high-fiber foods are considered the basis of a healthy dietary pattern with the potential benefit not only in preventing chronic diseases but also in ameliorating their course. Fiber intake recommendations range from around 20 to 38 g per day, depending on gender and age (21–25 g of fiber a day for women while 30–38 g a day for men) [43]. Nutrition is a key factor in cancer development, progression, and outcome of the therapy [44] and if meat, alcohol, salt preserved, and processed foods are considered as risk factors for different types of cancer [45], food rich in fibers (wholegrains, vegetables, legumes, and fruits) and fish are universally accepted to be healthy dietary patterns. Previous papers reported RS to lower the expression of oncogenic miR17-92 cluster, thus producing colorectal cancer-preventing effects [21,22]. Our study aimed to identify the effect of ERSD on miRNA expression profile in pancreatic cancer tissue of mice and the implication of miRNAs dysregulation on biological processes and cellular signaling pathways affected by RS, which could potentially benefit pancreatic cancer patients. We performed miRNA expression analysis on pancreatic cancer tissues from xenografted mice fed with high level of ERSD as compared to control diet and found almost 19 differentially expressed miRNAs (Figure 1A,B). By integrating this data with a public mRNA microarray dataset (GSE16515) [25] miRNA targets were predicted and miRNA-target genes pairs subsequently subjected to functional and pathways analyses. All these analyses displayed that the predicted miRNA-target genes associated to the higher levels of miRNA in tumor tissues from ERSD versus control fed mice were mainly involved in biological processes associated to synthesis of carbohydrate, glucose metabolism disorder, and cell death of cancer cell lines, as compared to mice fed with normal diet (Figure 2B and Table S2). Meanwhile, upregulated miRNA-target genes were associated to biological functions related to cancer, invasion, and metastasis of tumor, inflammatory response, and metabolic disease (Figure 2A,B and Table S2). Moreover, nine oncogenic pathways were overrepresented among these genes, including oncogenic—Neuregulin, Ephrin receptor, PI3K/AKT, and integrin signaling—and proinflammatory—IL-8, neuroinflammation, and NF-kB signaling—pathways, while the signaling of the tumor suppressor *PTEN* gene was enriched in pancreatic cancer tissues of mice fed with ERSD. In this regard, since we previously demonstrated a decrease in pancreatic cancer proliferation in mice fed with ERSD, as confirmed by a significantly lower expression of Ki67 at the mRNA and protein level [23], we further investigated the activation status of the proliferative PI3K/AKT pathway in tumors from both experimental groups, observing a decreasing trend upon ERSD. 

Expectedly, given the lower glycemic index of RS-enriched foods [46,47] carbohydrate metabolism and insulin signaling were also predicted to be altered upon ERSD.

Four out of the 19 miRNAs differentially expressed upon the two diets were found to have a prognostic value since, according to TGCA pancreatic cancer dataset, they were associated with an increased survival. This was the case of miR-375, miR-148a-3p, miR-125a-5p, and miR-200a-3p, all upregulated in mice fed with ERS diet with respect to the ones fed with control diet. These miRNAs associated with pancreatic cancer patients’ survival are known to be involved in cancer development and progression. miR-375 is frequently downregulated in pancreatic cancer tissues compared to normal tissues [31,38], was found associated with poor overall survival [35], and in vitro studies revealed its involvement in inhibiting cell proliferation, arresting cell cycle at G0/G1 phase, and inducing apoptosis of pancreatic cancer cells [38]. Additionally, the expression of miR-148a-3p was found lower in pancreatic cancer compared to normal tissues, and high expression of its target genes was associated with poor prognosis [33]. Moreover, in vitro experiments showed for this miRNA a proapoptotic action and inhibitory effects on migration and invasion on pancreatic cancer cells [33]. A tumor-suppressive role was described in cultured pancreatic cancer cells for miR-125a, which was shown to regulate energy metabolism, apoptosis, and migration [36]. Accordingly, previous studies already found a prognostic role for miR-125a, predicting a longer overall survival for pancreatic cancer patients [48,49]. Finally, miR-200a was found significantly downregulated in pancreatic cancer compared to paired normal control tissues [50]. Further, low levels of miR-200a were associated with poor pancreatic cancer differentiation and higher miR-200a expression in benign tissue predicted a longer relapse-free survival [34].

## 5. Conclusions

Dysregulation of miRNAs expression in pancreatic cancer could be associated with distinctive pathologic features and cancer behaviors. Overall, our results show that ERSD, as other nutrients, has the potential to modulate several biological molecules and pathways, potentially impacting the course of pancreatic cancer disease.

## Data Availability

The data presented in this study are available on request from the corresponding author and raw data are presented as Supplementary Table S1–S3.

## Supplementary Materials

The following are available online at https://www.mdpi.com/2218-273X/11/1/26/s1, Table S1: Differentially expression miRNA in ERSD vs. CTRL and their target genes predicted, Table S2: Ingenuity analysis of enrichment category for molecular and cellular functions of differentially expressed miRNA target genes.
